# Evolution to environmental contamination ablates the circadian clock of an aquatic sentinel species

**DOI:** 10.1002/ece3.3490

**Published:** 2017-10-28

**Authors:** Kayla D. Coldsnow, Rick A. Relyea, Jennifer M. Hurley

**Affiliations:** ^1^ Department of Biological Sciences Rensselaer Polytechnic Institute Troy NY USA; ^2^ Darrin Fresh Water Institute Rensselaer Polytechnic Institute Troy NY USA; ^3^ Center for Biotechnology and Interdisciplinary Studies Rensselaer Polytechnic Institute Troy NY USA

**Keywords:** entrained, external stimuli, pollution, road deicing salt, sodium–potassium pump, trophic cascade

## Abstract

Environmental contamination is a common cause of rapid evolution. Recent work has shown that *Daphnia pulex*, an important freshwater species, can rapidly evolve increased tolerance to a common contaminant, sodium chloride (NaCl) road salt. While such rapid evolution can benefit organisms, allowing them to adapt to new environmental conditions, it can also be associated with unforeseen tradeoffs. Given that exposure to environmental contaminants can cause circadian disruption, we investigated whether the circadian clock was affected by evolving a tolerance to high levels of road salt. By tracking the oscillations of a putative clock gene, *period*, we demonstrated that *D. pulex* express *per* mRNA with approximately 20‐hr oscillations under control conditions. This putative circadian rhythm was ablated in response to high levels of salinity; populations adapted to high NaCl concentrations exhibited an ablation of *period* oscillation. Moreover, we showed that while gene expression is increased in several other genes, including *clock*,* actin*, and *Na* ^*+*^ */K* ^*+*^ *‐ATPase*, upon the adaptation to high levels of salinity, *per* expression is unique among the genes we tracked in that it is the only gene repressed in response to salt adaptation. These results suggest that rapid evolution of salt tolerance occurs with the tradeoff of suppressed circadian function. The resultant circadian disruption may have profound consequences to individuals, populations, and aquatic food webs by affecting species interactions. In addition, our research suggests that circadian clocks may also be disrupted by the adaptation to other environmental contaminants.

## 1 INTRODUCTION

Anthropogenic disturbances such as habitat destruction and pollution events are rapidly changing the environment and threatening biodiversity worldwide (Dudgeon et al., 2006; Palumbi, 2001; Sih, Ferrari, & Harris, 2011). Changes in an ecosystem due to environmental contamination can induce rapid adaptation, which allows species to persist under contaminated conditions (Palumbi, 2001; Sih et al., 2011). However, these rapid adaptations often come with ecological tradeoffs, such as reduced reproduction, smaller body size, or increased susceptibility to other stressors (Ghazy, Habashy, Kossa, & Mohammady, 2009; Jansen, Stoks, Coors, van Doorslaer, & de Meester, 2011; Latta, Weider, Colbourne, & Pfrender, 2012). These tradeoffs can have profound consequences on individuals, populations, and entire ecological communities.

The salinization of freshwater habitats from sources like seawater intrusion, agriculture, mining, and road salt is an emergent anthropogenic disturbance threatening ecosystems worldwide (Cañedo‐Argüelles et al., 2013, 2016). In particular, road deicing salt application has increased from 0.20 million metric tons per year to 24.5 million metric tons per year in seven decades (Bolen, 2016; Harris & Tucker, 1947; Novotny & Stefan, 2010). Historically, most freshwater ecosystems have experienced low salinities (i.e., <20 mg Cl^−^/L; Kelting & Laxson, 2010). This suggests that freshwater ecosystems are vulnerable to increased salinities from road salt contamination. Thus, government agencies have set thresholds to protect organisms in these low‐salinity environments from increased salinity, such that the U.S. EPA has established chronic (230 mg Cl^−^/L) and acute (860 mg Cl^−^/L) thresholds (Benoit & Stephan, 1988). Unfortunately, an increasing number of freshwater ecosystems are experiencing salinities above these thresholds (Corsi, Graczyk, Geis, Booth, & Richards, 2010; Evans & Frick, 2001; Judd et al., 2005). For example, 51% of streams in the northern metropolitan U.S. exceeded the chronic threshold during colder months, and 15% exceeded the chronic threshold during warmer months (Corsi et al., 2010). This exemplifies that salinization is not simply a seasonal issue, but that soil and ground water contaminated with salt leach into systems year‐round, which demonstrates the pervasive impact of salinization (Jackson & Jobbágy, 2005; Van Meter & Swan, 2014).

Despite the vast extent of freshwater salinization, the full impact of salt contamination on freshwater communities has yet to be explored. A likely group affected by salinization is zooplankton. Studies show that zooplankton are some of the most sensitive organisms in freshwater communities and that many groups experience declines in abundance when exposed to increased salinities (Benoit & Stephan, 1988; Dalinsky et al., 2014; Dananay, Krynak, Krynak, & Benard, 2015; Evans & Frick, 2001; Hintz et al., 2017; Jones et al., 2017; Stoler et al., 2017; Van Meter & Swan, 2014; Van Meter, Swan, Leips, & Snodgrass, 2011). Zooplankton, especially the group *Daphnia,* are important in freshwater ecosystems due to their roles as primary feeders on phytoplankton and a preferred food source for many fish (Ebert, 2005; Seda & Petrusek, 2011). Often a decline in *Daphnia* results in increases in phytoplankton and a loss of ecosystem services (Carpenter, Kitchell, & Hodgson, 1985; Walsh, Carpenter, & Zanden, 2016). Therefore, to understand the effects of salinity increases on ecosystems, studies on the effects of salinization on species like *Daphnia* are vital.

While initial exposure to increased salinities can result in a decrease in *Daphnia* abundance, it can also result in sublethal consequences such as increased developmental abnormalities, reduced feeding rates, and decreased reproduction (Evans & Frick, 2001; Gonçalves, Castro, Pardal, & Gonçalves, 2007; Sarma, Nandini, Morales‐Ventura, Delgado‐Martínez, & González‐Valverde, 2006; Stoks, Geerts, & De Meester, 2014). *Daphnia* living in brackish environments (e.g., from close proximity to sea water) have evolved increased tolerance to salt (Ghazy et al., 2009; Latta et al., 2012). However, this tolerance is accompanied by tradeoffs, including smaller body size and reduced reproduction (Ghazy et al., 2009; Latta et al., 2012). Moreover, salt‐tolerant *Daphnia* also show differential expression of many genes, such as those responsible for Na^+^/K^+^‐ATPase pumps and predator sensing (Latta et al., 2012). Recent work has shown that *D. pulex* are able to rapidly evolve tolerance to the most common road salt, sodium chloride (NaCl; Coldsnow, Mattes, Hintz, & Relyea, 2017). This rapidly evolved tolerance could allow *D. pulex* to persist in aquatic communities exposed to anthropogenic increases in salinity, helping preserve water clarity, and maintain species interactions. However, the tradeoffs associated with this adaptation are unknown.

One potential tradeoff of evolved salt tolerance could be a disrupted circadian rhythm. Recent evidence shows that other environmental contaminants can disrupt circadian behavior/output, despite the circadian clock hypothetically being protected from environmental influences (Melvin, 2017; Numaguchi et al., 2015; Wang, Zhang, Xu, & Tischkau, 2014). Circadian clocks are endogenous molecular oscillators with a cycle near 24 hr (Panda, Hogenesch, & Kay, 2002). They control key biological processes that underlie a variety of daily functions, and chronic disruptions of the clock can have negative impacts on organismal biology (Evans & Davidson, 2013; Klarsfeld & Rouyer, 1998). We hypothesized that *Daphnia's* rapid adaptation to salinization could cause a disruption to the circadian system. Behavioral studies suggest *Daphnia* maintain a 24‐ to 28‐hr rhythm in constant conditions (Harris, 1963; Ringelberg & Servaas, 1971). Previous work utilizing the *D. pulex* genome established that many putative clock genes showed significant homology with known clock genes in *Drosophila*, a clock model organism (Allada & Chung, 2010; Tilden, McCoole, Harmon, Baer, & Christie, 2011). This conservation of genes implies that *D. pulex* maintain a clock with a similar architecture to well‐studied eukaryotic clocks (Bernatowicz et al., 2016; Tilden et al., 2011). In this study, we sought to determine whether *D. pulex* possesses a circadian rhythm and investigate whether rapid adaptation to increased salinization influenced the circadian clock.

## 2 MATERIAL AND METHODS

### 2.1 Strains

For all experiments, we used *D. pulex* originally collected from Northwest Bay, Lake George, USA. The *D. pulex* came from a previous food‐web experiment conducted during the summer of 2015 at the Rensselaer Aquatic Laboratory (Troy, NY, USA; (Hintz et al., 2017). The experiment exposed simple aquatic communities to five salt treatments (15, 100, 250, 500, 1,000 mg Cl^−^/L) in 1,200‐L outdoor mesocosms (Figure S1a,b) from 28 June to 22 September 2015. These mesocosms contained Lake George water (with a starting chloride concentration of 15 mg Cl^−^/L), sand substrate, and leaf litter. Each mesocosm also contained an aquatic food web comprised of the following: pouch snails (*Physa acuta*), amphipods (*Hyalella azteca*), isopods (*Asellus aquaticus*), banded mystery snails (*Viviparus georgianus*), fingernail clams (*Sphaerium simile*), phytoplankton, and zooplankton. The plankton, including *D. pulex*, were collected from various places throughout Northwest Bay, Lake George with a plankton tow (64‐micron mesh) and added to the mesocosms. To prevent organisms from departing or colonizing, each mesocosm was covered with 60% shade cloth. The salt treatments were applied on 10 July 2015 using Solar Salt (Morton^®^ Salt, Chicago, IL, USA), which is 99.8% pure sodium chloride (NaCl). Additional details of the food‐web experiment can be found in Hintz et al. (2017).

**Figure 1:**
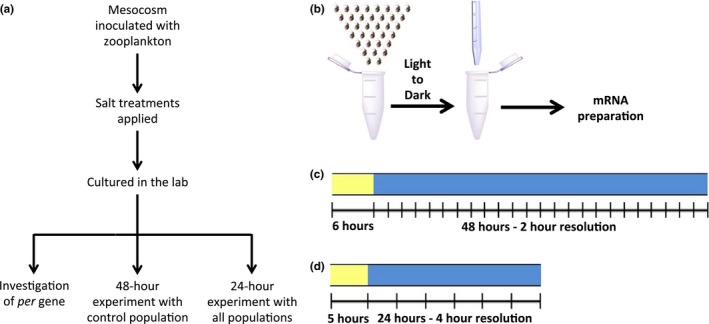
Experimental procedure for investigating the circadian rhythm of *Daphnia pulex*. (a) The order of events from the outdoor experiment to the indoor circadian rhythm experiments. Culturing time in the lab includes the time that *D. pulex* were removed from the mesocosms after living in various salt conditions and maintained in 15 mg Cl^−^/L water in the lab. (b) Experimental procedure for investigating the circadian rhythm. *Daphnia* photo credit: Mathew S. Schuler. (c) To demonstrate that *D. pulex* possess a molecular circadian clock*, Daphnia* were maintained in light for 6 hr before being transferred into the dark. These *Daphnia* were then sampled every 2 hr for 48 hr. (d) To investigate the consequences of chemical pollution, *Daphnia* were maintained in light for 5 hr before being transferred into the dark. These *Daphnia* were then sampled every 4 hr for 24 hr

Each mesocosm was sampled for *D. pulex* on 22 September using dip nets (100‐micron mesh). *D. pulex* populations were then isolated and cultured in the lab at constant temperature (20.5°C). The lab cultures were raised in Lake George water (15 mg Cl^−^/L) that had been filtered through glass microfiber filters (1.2‐μm pore size; Whatman, Inc.) to eliminate the addition of any other zooplankton. Each population from the food‐web experiment (15–1,000 mg Cl^−^/L) was raised separately at a density of approximately 40 large individuals/L. *D. pulex* were fed concentrated algae (*Psuedokirschneriella subcapitata*) that had been grown in COMBO media (Kilham, Kreeger, Lynn, Goulden, & Herrera, 1998) ad libitum every 2 days. Light regimes varied across the 8‐month husbandry stage. From September to early‐November 2015, zooplankton were raised under 12L:12D conditions. From late‐November 2015 to late‐February 2016, they were raised under 9L:15D. From March to May 2016, they were raised under 12L:12D. Tanks were cleaned and water was changed (using filtered Lake George water) as needed. In November 2015, the different strains of *Daphnia* were confirmed to have rapidly evolved tolerance to NaCl (Coldsnow et al., 2017). Because of *D. pulex*'s clonal nature, genetic differences between populations should have been conserved under these laboratory conditions. Additionally, pilot studies in January and March 2016 showed that the populations still differed in their salt tolerance. All strains were maintained in the lab until needed for the circadian experiments (Figure 1a). Additional information about the *Daphnia* strains can be found in Coldsnow et al. (2017).

### 2.2 Full circadian time course—48‐hr experiment

On 17 May 2016, we placed 35 *D. pulex* from the control (i.e., low‐salt) population (maintained in 15 mg Cl^−^/L) into 75 × 5 ml Eppendorf tubes with 5 ml of 15 mg Cl^−^/L water. We fed the *D. pulex* 5 hr prior to the start of the experiment (before they were separated into tubes). Lights were turned off at 13:00 EDT and this constituted the start of the experiment, which was defined as CT (circadian time) 12 and DD (hours in constant darkness) 0. Circadian time was calculated using the following equation: *(DD)(24‐hr period ÷ 20‐hr period) — CT12 ± 24 hr* (Dunlap & Loros, 2004). We then sampled the *D. pulex* in triplicate with a resolution of 2 hr over 48 hr starting with hour 0 (CT 12; DD 0). To sample at each time point, we removed all water and then placed the tube containing the sampled *Daphnia* into liquid nitrogen (Figure 1b,c).

### 2.3 Salt exposed populations—24‐hr experiment

On 6 January 2016, we collected 35 *D. pulex* from either a salt exposed *D. pulex* population (100, 250, 500, or 1,000 mg Cl^−^/L) or the control population (15 mg Cl^−^/L), all of which had been maintained in 15 mg Cl^−^/L. We placed the animals into 7 × 5 ml Eppendorf tubes that were filled with 5 ml of 15 mg Cl^−^/L water. We fed the *D. pulex* 4 hr prior to the start of the experiment (before they were separated into tubes). Lights were turned off at 12:00 EST, which was the start of the experiment (CT 12; DD 0). We then sampled each population (15, 100, 250, 500, or 1,000 mg Cl^−^/L) once every 4 hr for 24 hr starting with hour 0 (CT 12; DD 0). To sample at each time point, we removed all water from each tube and then placed the tubes containing the *Daphnia* into liquid nitrogen (Figure 1b,d).

### 2.4 RNA extraction and cDNA synthesis

After freezing in liquid nitrogen, we disrupted *D. pulex* cells using 1 ml of TRIzol^®^ Reagent (Life Technologies). We then pulverized each sample with a TissueLyser LT (Qiagen) and stored the resulting slurry at −80°C. Total RNA was extracted using the RNeasy mini kit (Qiagen) following established protocols (Lopez & Bohuski, 2007). We used the NanoDrop ND‐1,000 Spectrophotometer (NanoDrop Technologies) to check the quantity and quality of the extracted total RNA. cDNA synthesis was completed using SuperScript™ IV Reverse Transcriptase Kit (Invitrogen) and approximately 500 ng total RNA per sample.

### 2.5 Primer design and validation polymerase chain reaction

Primer 3 (version 0.4.0) was used to design primers to amplify the *period* gene (*per,* 322497), *clock* gene (*clk,* 346996), cytoplasmatic actin gene (*act1C*, 347742), and the gene encoding the alpha subunit of the Na^+^/K^+^‐ATPase pump (referred to here as *atp1A*, 309219). We used default settings and the last 500 bp of each gene (Table 1). Primers designed by Spanier et al. (2010) were used for the TATA‐binding protein (*tbp,* 194512*)* and syntaxin 16 (*stx16,* 194044*)* gene, which were used as reference genes (Table 1). For *per* and *tbp,* primer verification and the check for amplification in light and dark conditions were performed using standard polymerase chain reaction (PCR) techniques on cDNA prepared from *D. pulex* adapted to low‐salt conditions. This was done on a sample taken after 4 hr in the light, as well as on a sample taken after 16 hr in the dark. Amplified products using cDNA, primers (Integrated DNA Technologies), and OneTaq TM Hot Start polymerase (New England BioLabs) were run on a 1.8% agarose (IBI Scientific) gel.

**Table 1:** Target genes for PCR and qRT‐PCR

Gene name	Code	Putative function	Gene ID	L (aa)	Primer sequence (5′‐3′) ‐ Forward/Reverse	L (bp)	Source
Period	*per*	Sets cycle length	322497	1,233	TCGTCGAGAGATACGGATGA TTGTCCATCGGATTTTGTCA	152	This study
Clock	*clk*	Regulates circadian rhythms	346996	869	TCATTATGACGGCTGGTCAA AGGTCCCCAAGCTCCATACT	146	This study
Cytoplasmatic actin	*act1C*	Motility, intracellular transport.	347742	376	GCTCCATCCACCATGAAGAT TCCGGACTCGTCGTACTCTT	138	This study
Alpha subunit of Na^+^/K^+^‐ATPase	*atp1A*	Ion transport	309219	1,002	CGGCTGGTTTCTTCACCTAC GGAATCTGGGAGATCGTTGA	116	This study
TATA‐binding protein	*tbp*	Transcription initiation	194512	312	CTACGATGCATTCGATAACATATACC AGAACCAGCAATGAGTTAAACAAAG	144	Spanier et al. (2010)
Syntaxin 16	*stx16*	Exocytosis	194044	311	CACATTGGTCGTCCTTAGTCTTG TGCTATACGTTACGCTTGTCCTTAC	148	Spanier et al. (2010)

Code, Gene code; L (aa), Protein length; L (bp), Amplicon Length; PCR, polymerase chain reaction; qRT, quantitative real‐time.

### 2.6 qRT‐PCR

To quantitatively analyze all genes, approximately 50 ng of cDNA was amplified using QuantiFast SYBR Green (Qiagen) master mix, the respective primers, and the LightCycler^®^ 480 (Roche) for quantitative real‐time (qRT) PCR amplification. We performed qRT‐PCR using technical triplicates for each sample and the following settings: 5 min at 95°C; 35 cycles at 95°C for 10 s, followed by 60°C for 30 s. After completion of qRT‐PCR, we analyzed the output, or CT values.

### 2.7 Data analysis

For the 48‐hr experiment, we investigated the *per* and *tbp* genes. We averaged the CT values from the three technical replicates of the *per* and *tbp* gene. Using the equation 2^−ΔCT^ where $\Delta \text{CT}\;=\;\text{CT}\;{\text{value}}_{\mathrm{per}}-\text{CT}\;{\text{values}}_{\mathrm{tbp}}$, we calculated the expression levels of *per* relative to *tbp*. Biological replicates within an hour time point were averaged, and standard error of the mean (SEM) was obtained. To obtain the graph, we first normalized the data points by dividing by time point 0. The normalized averages were then graphed by both CT and DD. To create a trend line, a modified running average was used based on the average of a data point, the point before it, and the point after it. For the first data point, DD 0, 2 and 40 were used; for the last data point, DD 46, 48, and 6 were used; these time points represented the next closest CT time points. We used the JTK cycle package in R to obtain the period, amplitude, and lag (Hughes, Hogenesch, & Kornacker, 2010; R Core Team 2015).

For the 24‐hr experiment, we investigated the *per, clk, tbp,* and *stx16* genes for all populations. For the 15, 250, and 1,000 mg Cl^−^/L populations, we also investigated the *act1C* and *atp1A* genes. We averaged (AVG) the CT values from the three technical replicates for each gene and calculated the standard error of the mean (SEM). Within each time point and population, we calculated the geometric mean (GM) of two reference genes (*tbp* and *stx16)* and the propagation of error (POE) using the equation ${\text{POE}}_{\mathrm{ref}}=\frac{1}{2\mathrm{GM}}\sqrt{\frac{{{\text{AVG}}_{\mathrm{tbp}}}^{2}}{{\text{SEM}}_{\mathrm{tbp}}}+\frac{{{\text{AVG}}_{\mathrm{stx}16}}^{2}}{{\text{SEM}}_{\mathrm{stx}16}}}$ . We used two reference genes for the 24‐hr experiment since we were comparing different salt‐adapted populations and reference genes could be differentially affected by increases in salinity. Thus, averaging two would result in a better reference for qRT‐PCR (Vandesompele et al., 2002). Using the equation 2^−ΔCT^ where $\Delta \text{CT}\;=\;\text{CT}\;{\text{value}}_{\mathrm{target}}-{\text{GM}}_{\mathrm{ref}}$ *,* we calculated the expression levels of each target gene (*per, clk, act1C,* and *atp1A)* relative to the reference genes (*tbp* and *stx16)*. We also calculated the propagation of error (POE) by using the following equation: $\text{POE}=\ln\left(2\right)\sqrt{{\text{SEM}}_{\mathrm{target}}^{2}+{\text{POE}}_{\mathrm{ref}}^{2}}$. From here, we normalized the data points within a population by dividing by that population's time point 0. We then graphed the normalized data by both circadian time (CT) and hours in constant dark conditions (DD). Data points were connected because there was no replication, and therefore no biological variance in the data. All calculations were made with Microsoft Excel, and all graphs were made with DeltaGraph (version 5) for the 48‐ and 24‐hr experiments.

## 3 RESULTS

### 3.1 Determining a molecular circadian rhythm in *D. pulex*

**Figure 2:**
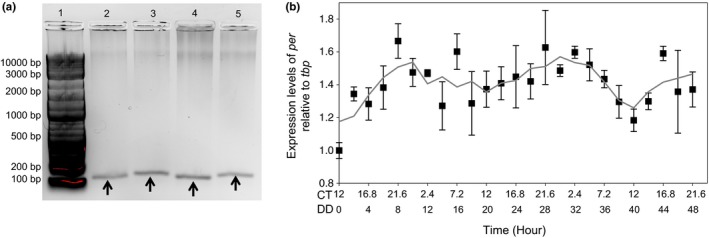
*Daphnia pulex* populations from low salt (15 mg Cl^−^/L) demonstrate a molecular circadian rhythm. (a) Successful amplification of *tbp* (lane 2) and *per* (lane 3) in the light, as well as *tbp* (lane 4) and *per* (lane 5) in the dark with a DNA ladder in lane 1. (b) The 48‐hr qRT‐PCR results for the control *D. pulex* population. All points are normalized with *tbp* and the first time point. The error bars indicate standard error between biological replicates that have been normalized with the first time point. The line represents a modified running average. The *x*‐axis has two scales: The first represents circadian time (CT) and the second represents hours in constant darkness (DD)

As the oscillations of the core clock gene *period* (*per*) are directly related to the period of the clock in *Drosophila,* we utilized the mRNA levels of the previously identified putative clock gene *per* as an output for clock function (Allada & Chung, 2010; Tilden et al., 2011; Tomioka & Matsumoto, 2010). We also selected the gene coding for the TATA‐binding protein (*tbp*) as a qRT‐PCR reference gene; its putative function suggested it was minimally regulated by the circadian clock (Spanier et al., 2010). To initially validate *per* and *tbp* expression, we extracted total cellular RNA from *D. pulex* cultured under light and dark conditions that were adapted to low levels (15 mg Cl^−^/L) of salt. The extracted RNA was reverse transcribed into cDNA, and then screened for *per* and *tbp* expression using primers targeted to the 3′ end of each respective gene (Table 1). We found that both primer sets amplified the desired product in the light sample (sampled after 4 hr of light exposure; Figure 2a, lanes 2 and 3) as well as the dark sample (sampled after 16 hr in the dark; Figure 2a, lanes 4 and 5), demonstrating that *per* and *tbp* were expressed under our culture conditions.

Recent work suggests a circadian oscillation in *D. pulex* on the molecular level (Bernatowicz et al., 2016; Rund et al., 2016). However, a molecular clock in *D. pulex* has not been shown to persist without external cues, which is a requirement of endogenous clocks. Therefore, to study whether the circadian clock of *D. pulex* is affected by the adaptation to the environmental contaminant road salt, we first needed to demonstrate that the proposed circadian rhythm in *Daphnia* was a bona fide circadian rhythm. To do so, we maintained the control, low‐salt (15 mg Cl^−^/L) *D. pulex* adapted strain in DD (constant darkness) conditions and sampled in triplicate at a 2‐hr resolution for 48 hr. For each sample, we extracted total mRNA, reverse transcribed the mRNA, and then performed qRT‐PCR (see Experimental Procedure; Figure 1b,c). The control, low‐salt *D. pulex* population demonstrated that *per* mRNA levels (relative to *tbp)* oscillated two full cycles over a 48‐hr period (Figure 2b). The period length of *per* mRNA was determined by JTK_cycle to be approximately 20 hr with a lag of 11 hr and amplitude of 0.163 (*p* = .018) (Hughes et al., 2010). The expression of *per* mRNA peaked at CT (circadian time) 24 (DD 10 and DD 30) with troughs at CT 12 (DD 20 and 40).

### 3.2 Determining whether evolved salt tolerance disrupts the circadian clock in *D. pulex*

As the adaptation to environmental contamination is often accompanied by tradeoffs and environmental contaminants can disrupt circadian behavior/output (Melvin, 2017; Numaguchi et al., 2015; Wang et al., 2014), we investigated whether evolved salt tolerance could affect the circadian clock of *D. pulex*. We examined the mRNA expression levels of *per* and an additional putative clock gene, *clock (clk)*, in five *D. pulex* populations adapted to a wide range of salinities (15–1,000 mg Cl^−^/L) (Coldsnow et al., 2017; Hintz et al., 2017). *per* and *clk* are both key players in the circadian transcription–translation negative feedback loop. *clk* is the activator of *per* transcription and *per* represses *clk* activity, and therefore its own transcription (Allada & Chung, 2010; Tomioka & Matsumoto, 2010). For each population, we utilized the same techniques described above to determine the levels of *per* and *clk* mRNA from *D. pulex*, this time sampling over 24 hr with a 4‐hr resolution and using two reference genes, *tbp* and *syntaxin 16* (*stx16;* see Experimental Procedure; Figure 1b,d).

**Figure 3:**
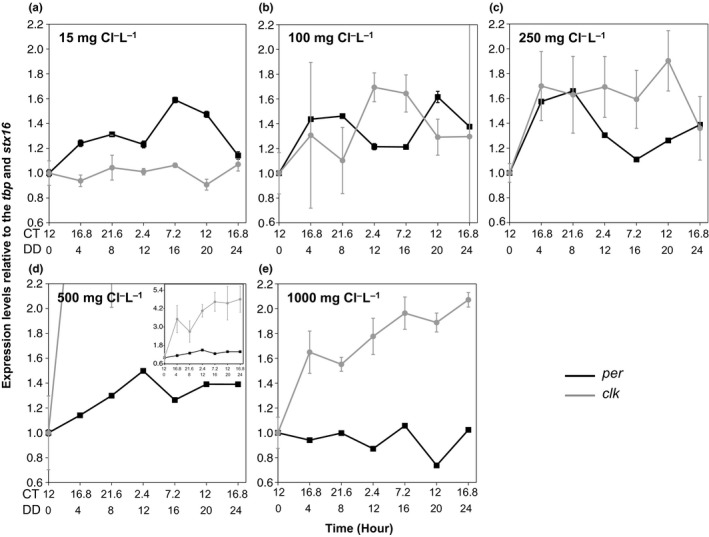
The adaptation to high salt concentrations disrupts the circadian rhythm in *Daphnia pulex*. The 24‐hr qRT‐PCR results for: (a) 15 mg Cl^−^/L (control), (b) 100 mg Cl^−^/L, (c) 250 mg Cl^−^/L, (d) 500 mg Cl^−^/L, and (e) 1,000 mg Cl^−^/L populations. All data points have been normalized with the geometric mean of *tbp* and *stx16,* and the first time point. The error bars indicate propagation of error between the technical qPCR replicates normalized with the first time point. All points have error bars; some are masked by the data point. The *x*‐axis has two scales: The first represents circadian time, (CT) and the second represents hours in constant darkness (DD)

*per* expression levels (relative to *tbp* and *stx16*) in the control population (15 mg Cl^−^/L) increased upon transition from LL (constant light) to DD, showing a slight bimodal curve and reaching a peak around CT 7.2 (DD 16; Figure 3a). *clk* expression levels (relative to *tbp* and *stx16*) in the control population did not increase upon transition from LL to DD, but rather had a slight oscillation over the 24‐hr experiment (Figure 3a). At this resolution, we could not accurately estimate a period. Therefore, to determine the effect of salt adaptation on the circadian clock, we instead compared each salt‐adapted population to this control population (15 mg Cl^−^/L).

Similar to the control population, *per* expression levels in populations adapted to moderate salinity concentrations (100 and 250 mg Cl^−^/L) increased upon transition to DD, but have important differences (Figure 3a–c). Most notably, *per* expression levels in the 100 mg Cl^−^/L population showed a more pronounced bimodal curve, peaking between CT 16.8 and 21.6 (DD 4 and 8) and then again at CT 12 (DD 20; Figure 3b). *per* expression levels in the 250 mg Cl^−^/L population showed the highest peak around CT 21.6 (DD 8; Figure 3c), but showed a large trough at CT 7.2 (DD 7.2) and no additional peaks. Unlike *clk* in the control strain, *clk* expression levels in the populations adapted to moderate salinity showed a more robust oscillation over the 24 hr (Figure 3a–c). In both the 100 and 250 mg Cl^−^/L populations, *clk* expression levels increased after the LL to DD transition and showed an antiphase relationship with *per*, peaking between CT 2.4 and 7.2 (DD 12 and 16; Figure 3b,c). Together, the moderate salt‐adapted populations show changes in both *per* and *clk* expression levels, and our data suggest either a period or phase difference in the clock between the control populations and the populations adapted to moderate salinity concentrations.

Notably, there were substantial differences in the 500 and 1,000 mg Cl^−^/L adapted populations when compared to the 15 mg Cl^−^/L population (Figure 3a,d,e). While *per* expression levels in the 500 mg Cl^−^/L population increased when placed into DD, it showed no circadian pattern or oscillation. Contrary to all other populations, *per* expression levels in the 1,000 mg Cl^−^/L population decreased when placed into DD conditions and like the 500 mg Cl^−^/L population, appeared to have no oscillations in *per* levels (Figure 3e). *clk* in the 500 and 1,000 mg Cl^−^/L populations showed large and continuous increases in *clk* expression upon the LL to DD transition (Figure 3e). Together, the high‐salt‐adapted populations show drastic changes in core clock gene expression levels. In general, the expression levels of *per* and *clk* in all populations showed an inverse relationship to each other and when oscillations occurred, displayed an antiphasic relationship, likely a consequence of the negative feedback loop.

### 3.3 Determining whether evolved salt tolerance disrupts other essential functions in *D. pulex*

To investigate the expression levels of other important *Daphnia* genes in populations adapted to high salinity, we tracked the expression of two significant *Daphnia* genes, *cytoplasmatic actin* (*act1C*) and the gene encoding the alpha subunit of the Na^+^/K^+^‐ATPase pump (referred to here as *atp1A*). Actin is a vital protein involved in cellular motility, cell division, muscular movement, and intracellular transport; actin gene expression is often used as a control gene in qPCR analyses. (Cooper, 2000). Na^+^/K^+^‐ATPases are major transporter proteins that respond to salinity increases and are thought to be responsible for increased salt tolerance (Kefford et al., 2016). Importantly, *atp1A* is upregulated in salt‐tolerant *Daphnia* populations (Latta et al., 2012). Moreover, changes to this gene are accommodated into the genome, whether epigenetic or genetic, such that changes may persist when the stressor is gone and may be transferred from generation to generation, as opposed to plastic, which is the case for other subunits of Na^+^/K^+^‐ATPases (Latta et al., 2012). We examined the levels of *act1C* and *atp1A* mRNA in the 15, 250, and 1,000 mg Cl^−^/L populations, representing a low (control), moderate, and high salinity population. The mRNA came from the above‐mentioned 24‐hr, 4‐hr resolution experiment (see Experimental Procedure; Figure 1b,d).

**Figure 4:**
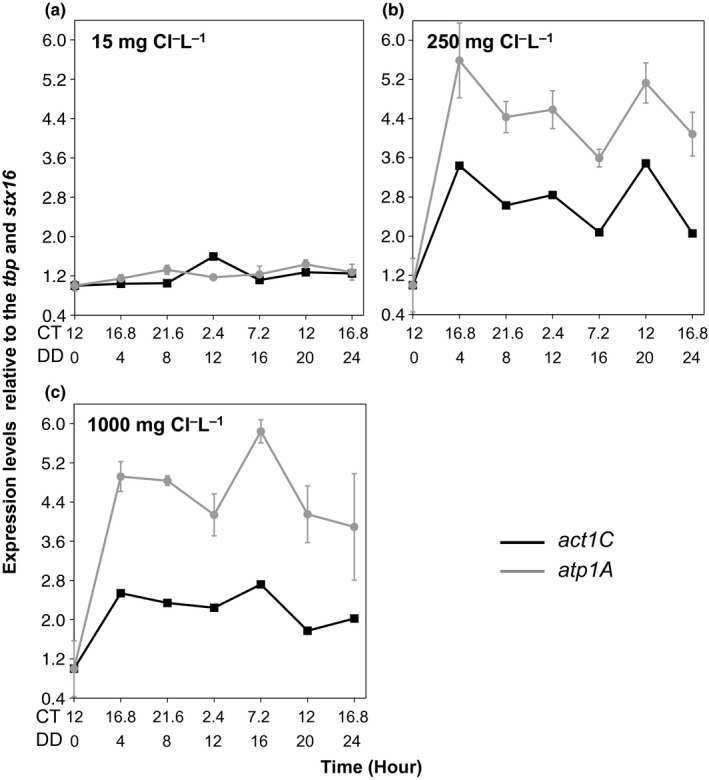
The adaptation to high salt concentrations disrupts vital genes. The 24‐hr qRT‐PCR results for (a) 15 mg Cl^−^/L (control), (b) 250 mg Cl^−^/L, and (c) 1,000 mg Cl^−^/L populations. All data points have been normalized with the geometric mean of *tbp* and *stx16,* and the first time point. The error bars indicate propagation of error between the technical qPCR replicates normalized with the first time point. All points have error bars; some are masked by the data point. The *x*‐axis has two scales: The first represents circadian time, (CT) and the second represents hours in constant darkness (DD)

*act1C* and *atp1A* expression levels in the control population remained relatively constant and showed little increase upon the LL to DD transition, though *act1C* did show a small peak at CT 2.4 (DD 12; Figure 4a). In the 250 and 1,000 mg Cl^−^/L population, however, *act1C* and *atp1A* expression levels showed sharp increases upon the LL to DD transition, especially the expression levels of *atp1A*. For both salt‐adapted populations, there were multiple peaks and troughs in the expression levels of *act1C* and *atp1A*.

## 4 DISCUSSION

A basic tenant of an endogenous circadian clock is that it must persist in the absence of external cues (Hurley, Loros, & Dunlap, 2016). Therefore, for *D. pulex* to have a functioning clock, *per* expression levels should oscillate in a circadian manner in the absence of light/dark cycles. We showed that *per* expression levels oscillate over 48 hr in complete darkness with a 20‐hr period, validating that *D. pulex* maintain an endogenous circadian rhythm. Further, our data corroborate more recent work that shows that *D. pulex* have expression of core clock genes (Bernatowicz et al., 2016; Rund et al., 2016). Bernatowicz et al. (2016) and Rund et al. (2016) also show an increase in *per* expression levels at approximately CT 12, or the beginning of the subjective night, as well as minimal oscillations in the *clk* gene. Though there are differences between these studies’ results and our own, these differences could be attributed to disparities in many key areas, including husbandry, experimental light conditions (constant dark vs. light–dark cycles), experimental duration (24 vs. 48 hr), experimental approach, and differences in the *Daphnia* clones. In conjunction with the conservation of core clock genes, our data suggest that *D. pulex* maintain a molecular clock with similar architecture to those studied in other eukaryotic organisms (Partch, Green, & Takahashi, 2014; Tilden et al., 2011). Of note, the expression levels of *per* and *clk* in our control population closely resemble the expression of these genes in mosquitoes (*Anopheles gambiae*), relevant as the circadian rhythm of *D. pulex* is thought to be more like that of butterflies and mosquitos than that of *Drosophila* (Bernatowicz et al., 2016; Meireles‐Filho & Kyriacou, 2013; Rund et al., 2016; Tilden et al., 2011).

Studies on circadian disruption due to pollutant exposure are limited primarily to the effects of light pollution and the mechanistic underpinnings of these effects are well characterized (Chepesiuk, 2009). Other studies have begun to connect organismal exposure to other common pollutants to circadian disruption, such as antidepressants, dioxins, and cigarette smoke, which can have consequences on the phase and period of cyclic behavior and circadian output (Melvin, 2017; Numaguchi et al., 2015; Wang et al., 2014). However, our work is important as it is the first to demonstrate a direct effect of evolved tolerance to an environmental contaminant on the core molecular oscillator, showing that adaptation to road salt drastically affects the molecular clock in *D. pulex*. The adaptation of *D. pulex* to moderate concentrations of NaCl road salt resulted in moderate differences in *per* and *clk* expression, potentially a consequence of a phase shift or a shortened period. Remarkably, the adaptation of *D. pulex* to high concentrations of NaCl road salt resulted in the ablation of *per* oscillation and an increase in *clk* expression, potentially a consequence of *per* gene suppression. However, changes in *per* and *clk* gene expression could also be an indirect result of gene expression at multiple points in the circadian negative feedback loop, as many different proteins are involved in gene regulation in insect clocks (Allada & Chung, 2010; Tomioka & Matsumoto, 2010). Additionally, some differences may be explained by the use of whole‐body mRNA versus mRNA extracted from the head, and further research should investigate circadian rhythm disruptions in specific organs.

While we showed that the evolution of salt tolerance in *D. pulex* disrupts genes we presume to be vital to the circadian clock, we also showed that alterations in gene expression are not circadian specific. *atp1A* showed substantial changes in salt‐tolerant populations when compared to the control, low‐salt population. In addition, other studies show that salinity changes can result in changes to Na^+^/K^+^‐ATPases, as they are thought to be involved in salt acclimation and adaptation (Ituarte, Mañanes, Spivak, & Anger, 2008; Latta et al., 2012; Wu, Yang, Lee, Gomez‐Mestre, & Kam, 2014). We also showed changes to *act1C,* a gene coding for the actin protein. It is likely that many other genes are also affected since Latta et al. (2012) showed that close to 500 genes are differentially regulated in naturally salt‐tolerant *Daphnia* populations and that subsequent exposure to increased salinities can alter that number. Therefore, there may be widespread changes to many other gene expression levels in *Daphnia* populations adapted to increased levels of NaCl road salt. However, we note that of the limited number of genes that we tested, the core clock gene *per* was the only gene that underwent a suppression of expression (Figure 3).

The profound questions implicated by this work are two‐fold: (1) What is the mechanistic underpinning of the inhibition of clock gene expression? (2) What are the ecological consequences of circadian disruption? While the mechanism remains unknown, previous studies have shown a significant increase in global DNA methylation in *D. magna* exposed to NaCl*,* suggesting that there may be widespread epigenetic changes upon exposure to increased salinity (Asselman et al., 2015). Additional work has demonstrated significant differences in the expression levels of many genes between salt tolerant and intolerant *D. pulex* (Latta et al., 2012). This indicates that epigenetic changes may be involved in the evolution of salt tolerance and, by extension, the disruption of the circadian clock. In this study, we see a graded effect on expression levels relative to NaCl concentration; further evidence that epigenetic changes could be responsible.

Importantly, the clock appears to be conserved between *D. pulex* and higher eukaryotes. Further, *Daphnia* are a common model for how organisms respond to environmental stress. Therefore, it is possible that the impacts on the circadian clock due to environmental exposure may also be conserved between *Daphnia* and higher eukaryotes. This suggests that higher eukaryotic clocks, such as those found in humans, may be affected by the adaptation to pollutants at the level of the core oscillator. As epigenetic changes can occur quickly, well within organismal lifespans, it is therefore important to understand the mechanistic underpinning of circadian rhythm disruption due to the adaptation to environmental contamination because these effects may have a direct societal impact. Our work also suggests that *D. pulex* may serve as a good model system to determine how the human clock adapts to environmental pollutants (Colbourne et al., 2011).

In regards to the ecological consequences of circadian disruption, an ablated circadian rhythm could affect reproduction, growth, longevity, immune function, or other behaviors (Evans & Davidson, 2013; Karatsoreos, Bhagat, Bloss, Morrison, & McEwen, 2011). One important behavior of *Daphnia* (and many other zooplankton) is diel vertical migration (DVM), which is a daily mass movement of zooplankton worldwide (Brierley, 2014). During DVM, organisms travel to the surface at night to feed and then migrate back down beneath the photic zone during the day to avoid visually hunting predators (Brierley, 2014; Ebert, 2005). While it is debated whether a circadian clock, external cues (e.g., predators, sunlight), or some combination controls DVM (Cohen & Forward, 2009; Williamson, Fischer, Bollens, Overholt, & Breckenridge, 2011) and many studies find that external cues influence DVM, the biological clock is suggested to play a role in the entrainment and regulation of the system. Therefore, via disruption of DVM through clock misregulation, the adaptation to salinization could cause changes in zooplankton community composition, abundance, or behavior. This could have detrimental effects ranging from phytoplankton blooms to decreases in top predators (Carpenter et al., 1985; Walsh et al., 2016). These changes can lower water quality and cause food shortages for organisms, including humans (Walsh et al., 2016).

As we are now living in what some call the Anthropocene, organisms including humans are increasingly exposed to a variety of contaminants. Road salt is a common environmental contaminant and our work suggests that circadian disruption caused by the adaptation to salinization in *D. pulex,* and possibly other organisms, may be pervasive and increasing (Cañedo‐Argüelles et al., 2013, 2016). Other contaminants like pesticides, pharmaceuticals, and heavy metals are also becoming increasingly common in fresh water (Pal, Gin, Lin, & Reinhard, 2010; Warren, Allan, Carter, House, & Parker, 2003) and similarly, organisms are known to adapt to these contaminants as well (Bendis & Relyea, 2014; Hochmuth, De Meester, Pereira, Janssen, & De Schamphelaere, 2015; Hoy, 1998). The tradeoffs of these adaptations, however, remain unknown and have largely not been investigated. This suggests that our research demonstrating circadian rhythm disruption from chemical contamination and its proposed physiological, psychological, and behavioral consequences may just be the tip of the proverbial iceberg.

## DATA ARCHIVAL LOCATION

Data will be placed on Dryad doi:10.5061/dryad.761k6.

## CONFLICT OF INTEREST

None declared.

## AUTHOR CONTRIBUTIONS

KDC and JMH designed the experiments. KDC conducted the experiments, analyzed the results, and wrote the original draft. JMH provided guidance. KDC, JMH and RAR reviewed and edited the paper. JMH and RAR provided funding and resources.
