# Supplementary material for: Evolution to environmental contamination ablates the circadian clock of an aquatic sentinel species
Source: Ecol Evol. 2017 Oct 28;7(23):10339–49. doi: 10.1002/ece3.3490 (PMC5723614; doi:10.1002/ece3.3490)
Supplement: Supplementary file 1 [file ECE3-7-10339-s001.pdf]

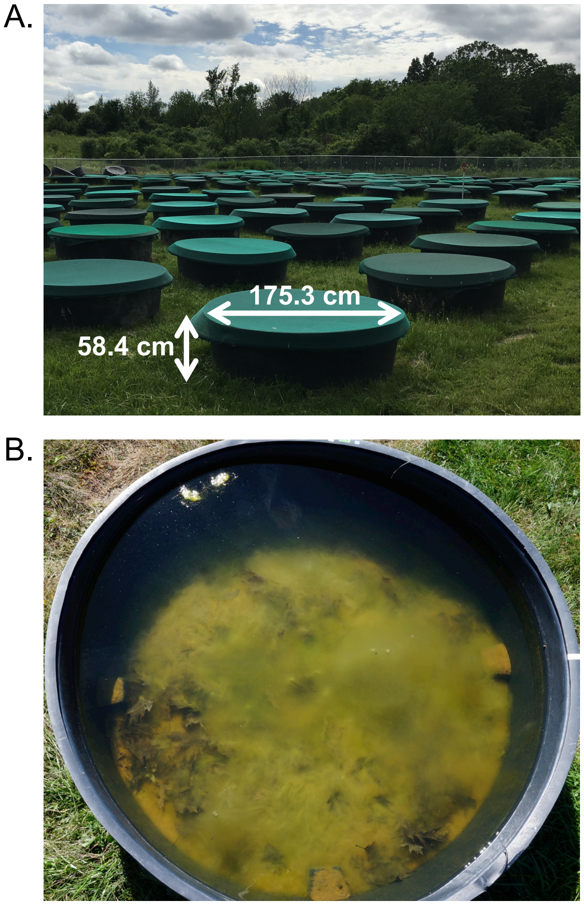

Supplemental Figure 1. The experimental set up for the evolution of salt tolerance in *D. pulex* (A) The outdoor experiment involving 1200-L cattle tanks (mesocosms). (B) An example of a mesocosm that contains control *D. pulex* raised in 15 mg Cl^{-}/L Lake George water. Photo credit: William D. Hintz.
